# Umbilical cord blood-derived mesenchymal stem cells consist of a unique population of progenitors co-expressing mesenchymal stem cell and neuronal markers capable of instantaneous neuronal differentiation

**DOI:** 10.1186/scrt148

**Published:** 2012-12-19

**Authors:** Mundackal S Divya, George E Roshin, Thulasi S Divya, Vazhanthodi Abdul Rasheed, Thankayyan R Santhoshkumar, Kandathil E Elizabeth, Jackson James, Radhakrishna M Pillai

**Affiliations:** 1 Neuro-Stem Cell Biology Laboratory, Department of Neurobiology, Rajiv Gandhi Centre for Biotechnology, Thiruvananthapuram, Kerala 695 014, India; 2 Sree Avittom Thirunal Hospital for Women & Children, Government Medical College, Thiruvananthapuram, Kerala 695 011, India; 3 Cancer Research Program, Rajiv Gandhi Centre for Biotechnology, Thiruvananthapuram, Kerala 695 014, India

## Abstract

**Introduction:**

Umbilical cord blood (UCB)-derived mesenchymal stem cells (MSCs) are self-renewing multipotent progenitors with the potential to differentiate into multiple lineages of mesoderm, in addition to generating ectodermal and endodermal lineages by crossing the germline barrier. In the present study we have investigated the ability of UCB-MSCs to generate neurons, since we were able to observe varying degrees of neuronal differentiation from a few batches of UCB-MSCs with very simple neuronal induction protocols whereas other batches required extensive exposure to combination of growth factors in a stepwise protocol. Our hypothesis was therefore that the human UCB-MSCs would contain multiple types of progenitors with varying neurogenic potential and that the ratio of the progenitors with high and low neurogenic potentials varies in different batches of UCB.

**Methods:**

In total we collected 45 UCB samples, nine of which generated MSCs that were further expanded and characterized using immunofluorescence, fluorescence-activated cell sorting and RT-PCR analysis. The neuronal differentiation potential of the UCB-MSCs was analyzed with exposure to combination of growth factors.

**Results:**

We could identify two different populations of progenitors within the UCB-MSCs. One population represented progenitors with innate neurogenic potential that initially express pluripotent stem cell markers such as Oct4, Nanog, Sox2, ABCG2 and neuro-ectodermal marker nestin and are capable of expanding and differentiating into neurons with exposure to simple neuronal induction conditions. The remaining population of cells, typically expressing MSC markers, requires extensive exposure to a combination of growth factors to transdifferentiate into neurons. Interesting to note was that both of these cell populations were positive for CD29 and CD105, indicating their MSC lineage, but showed prominent difference in their neurogenic potential.

**Conclusion:**

Our results suggest that the expanded UCB-derived MSCs harbor a small unique population of cells that express pluripotent stem cell markers along with MSC markers and possess an inherent neurogenic potential. These pluripotent progenitors later generate cells expressing neural progenitor markers and are responsible for the instantaneous neuronal differentiation; the ratio of these pluripotent marker expressing cells in a batch determines the innate neurogenic potential.

## Introduction

Umbilical cord blood (UCB) is considered one of the most abundant sources of non-embryonic stem cells [1]. The collection of mesenchymal stem cells (MSCs) from UCB that is discarded at the time of birth is an easier, less expensive and non-invasive method than collecting MSCs from bone marrow aspirates [2]. These MSCs attract special interest due to these specific advantages over embryonic and adult stem cell counterparts, since there are also no ethical issues associated with UCB. Another important characteristic of UCB-MSCs is that they are less immunogenic, and therefore do not elicit the proliferative response of allogeneic lymphocytes *in vitro *[3]. UCB-MSCs expanded *in vitro *also retain low immunogenicity and an immunomodulatory effect. Moreover, cells derived from the UCB elicit a lower incidence of graft rejection and post-transplant infections compared with other sources [4].

Considering these facts, UCB-derived MSCs can therefore be effectively utilized for therapeutic applications of various diseases. These applications include cell-based therapy to replenish degenerated neurons, cardiac cells, muscle cells, chondrocytes, and so forth. However, the potential application of these cells for various purposes requires extensive characterization, and requires standardization of reproducible differentiation protocols with ultimate functional characterization of the differentiated cells. Morphologically, the MSCs are adherent, fibroblast-like cells [5] with multipotent differentiation potential and thus can be induced to differentiate into cells of multiple lineages such as adipocytes, osteocytes, chondrocytes, myocytes, hepatocytes, neurons and astrocytes [6-10]. Several groups have explored the possibility of generating functional neurons from UCB-MSCs to use for various neurodegenerative diseases. Administration of human umbilical cord blood **(**hUCB)-MSCs was found to be feasible treatment for brain injuries such as stroke and other degenerative disorders [11,12]. Transplanting hUCB-MSCs in spinal cord injury animal models has shown significant improvement in neurological function [13].

Even though the hUCB-derived cells have been shown to differentiate into different lineages [14], a high potential for neuronal differentiation has been shown with extensive exposure to multiple combinations of growth factors [15-18]. *In vitro *treatment with β-mercaptoethanol and retinoic acid resulted in a very drastic difference in cellular morphology of MSCs from fibroblastic to spindle-shaped with elongated processes resembling a neuronal phenotype [19]. Previous reports have shown that hUCB-MSCs can be transdifferentiated into neuronal lineage by treating with nerve growth factor and retinoic acid. This multi-lineage differentiation capacity, the expression of neural properties and overlapping genetic programs for hematopoiesis and neuropoiesis [20] suggest that hUCB cells may have the ability to transdifferentiate in to neural cells. Few reports have shown that UCB-derived progenitors can express Oct3/4, Sox2, Nanog and Rex1, which are pluripotent/multi-lineage markers and could possibly differentiate into multiple lineages [21-23]. Convincing evidence therefore appears to show that hUCB-MSCs can be used as a good source for generating neurons, but there exists a considerable difference in the neurogenic potential of different batches of MSCs obtained from UCB.

The multi-lineage differentiation capacity and an inherent neuronal differentiation potential observed in a few batches made us characterize the hUCB-derived cells in detail with respect to neurogenic potential, since evaluating the neurogenic potential of hUCB-MSCs isolated for any possible cell replacement therapeutic applications is really important. Our results have in fact shown that the hUCB-MSCs varied in their neurogenic differentiation potential between samples, with a few showing the presence of a unique population of cells with inherent neurogenic potential even though they phenotypically express MSC markers.

## Materials and methods

### Umbilical cord blood MSC isolation and culture

UCB samples were collected from 45 full-term delivery cases with previous consent from the mothers according to the Institute's human ethical committee guidelines. The UCB samples collected were coded as hUCB-MSC SCB1 to SCB45. The samples used for mononuclear cell (MNC) isolation alone were coded as hUCB MNC01 to MNC03. Clinical parameters for the mother and baby were also noted for latter correlation with the yield of MSCs. The parameters recorded were the gestation period, UCB blood volume collected, and body weight of the baby (see Table S1 in Additional file 1).

UCB units were collected into 50 ml sterile tubes containing anticoagulant (acid citrate dextrose solution). All samples were processed within 4 to 6 hours from collection. Blood was diluted in 1× PBS in a 1:1 ratio and MNCs were isolated by density gradient centrifugation using Percoll gradient (GE Life Sciences, Piscataway, NJ, USA) [24]. MNCs obtained after the Percoll gradient separation were washed twice in 1× PBS and resuspended with CD34 antibody-conjugated beads (Miltenyi Biotech, Cologne, Germany) and were incubated for 30 minutes at 4°C followed by selective depletion of CD34-positive cells. The CD34-depleted population was collected and further resuspended in proliferation medium consisting of Iscove's modified Dulbecco's medium (IMDM) (Gibco, Life Technologies, Carlsbad, CA, USA) with 20% FBS (Hyclone Laboratories, Logan, UT, USA), 20 ng/ml fibroblast growth factor (FGF)-2 (Chemicon, Millipore, Billerica, MA, USA), 2 mg/ml heparin (Sigma, St.Louis, MO, USA), 1% penicillin/streptomycin (Gibco) and was plated in T25 flasks at a density of 5 × 10^6 ^cells/ml. After 24 hours of incubation at 37°C with 5% CO_2_, the nonadherent cells were washed off and the attached cells were expanded further with medium being replenished every alternate day. All differentiation protocols were carried out with MSCs between passages 3 and 4. Immortalized hUCB-derived MSC line RCB 2080 procured from RIKEN (Tsukuba, Ibaraki, Japan) was cultured in the same medium and was used as a control. Cells were passaged upon reaching 70 to 80% confluence using 0.05% Trypsin-ethylenediamine tetraacetate (Gibco) and were replated or cryopreserved for subsequent experiments.

### Immunophenotyping of human umbilical cord blood MSCs

Characterization of hUCB-MSCs was carried out by immunophenotyping using both MSC-positive and MSC-negative surface markers. Briefly, 60 to 80% confluent flasks of expanded MSCs were trypsinized, followed by washing with 1× PBS and fixed in 4% paraformaldehyde for 15 minutes at 4°C. Cells were then incubated with FITC/PE-conjugated CD73 (1:100; BD Pharmingen, San diego, CA, USA), CD44 (1:100; BD Pharmingen), CD45 (1:100; BD Pharmingen), CD105 (1:100; BD Pharmingen) and CD29 (1:100; BD Pharmingen) primary antibodies in the dark at 4°C for 1 hour and finally resuspended in 1× PBS containing 3% BSA for fluorescence-activated cell sorting analysis. To avoid nonspecificity and background staining, appropriate isotype secondary antibody controls (mouse IgG-Att488 and mouse IgG-PE) and cell-only controls were used. A total of 10,000 events were analyzed using a BD FACS Aria Flow Cytometer (BD Biosciences, San jos, CA, USA).

### Immunofluorescence analysis

For immunostaining, hUCB-MSCs were grown on coverslips in 24-well plates for 48 hours for proliferation and for 5 to 12 days for differentiation. Immunofluorescence analysis of cells was carried out as described previously [25]. Briefly, cells were fixed in 4% paraformaldehyde (Sigma), followed by blocking in 0.4% blocking solution for detecting nuclear antigens,0.2% blocking solution for cytoplasmic antigens and 0.1% blocking solution for surface antigens. The cells were then incubated with the following primary antibodies against β-III-tubulin (1:200; Chemicon), nestin (1:50; Developmental Studies Hybridoma Bank, University of Iowa, Iowa City, Iowa, USA), 5-bromo-2-deoxyuridine (BrdU) (1:100; Abcam Cambridge, UK), Sox2 (1:500; Abcam), Musashi (1:100; Chemicon), Vimentin (1:100; Developmental Studies Hybridoma Bank), CD105 (1:100; BD Pharmingen), CD29 (1:100; BD Pharmingen), CD44 (1:100; BD Pharmingen), CD73 (1:100; BD Pharmingen) and CD45 (1:100; BD Pharmingen). This incubation was followed by nuclear staining with 4',6-diamidino-2-phenylindole (DAPI) (Sigma).

Cells were examined for epifluorescence following incubation with appropriate secondary antibody conjugated to Cy3/FITC (1:400; Jackson Immunoresearch, Philadelphia, PA, USA) in an upright fluorescent microscope (Olympus BX61, Olympus, Tokyo, Japan) and images were captured using a cooled CCD camera (Andor 885; Andor Technologies, Belfast, UK). For quantification of the percentage of cells expressing a specific marker in any given experiment, the number of positive cells of the whole population was determined relative to the total number of 4',6-diamidino-2-phenylindole-stained cells. The specificity of nestin, Sox2 and Musashi antibodies were determined using embryonic day 14 cortical neuronal cultures (see Figure S1 in Additional file 2); similarly, nonspecific binding of flurochrome-conjugated secondary antibodies was excluded by incubating the secondary antibody alone with MSC cultures (see Figure S2 in Additional file 3).

### 5-Bromo-2-deoxyuridine incorporation and proliferation assay

BrdU incorporation for proliferation analysis was performed as previously described [26]. Briefly, 10 μM BrdU (Sigma) was added along with culture medium for proliferation assays. Cells were fixed in 4% paraformaldehyde at 4°C for 15 minutes followed by treatment with 2 N HCl for 45 minutes and with 0.1 M boric acid for 10 minutes to expose the DNA for BrdU immunostaining. The treated cells were incubated with primary anti-BrdU antibody (1:100; Abcam) overnight at 4°C followed by 1 hour of incubation at room temperature with goat anti-rat FITC-conjugated secondary antibody.

### RT-PCR analysis

RT-PCR analysis was carried out as previously described [25]. Briefly, total RNA was isolated from unexpanded hUCB-MNCs, proliferating UCB-MSCs and differentiating UCB-MSCs using an RNA isolation kit (Qiagen RNAeasy kit, Qiagen, Valencia, CA, USA). Then ~2 μg RNA was transcribed into cDNA using random hexamers (Promega Corporation, Madison, WI, USA) and Superscript RT (Invitrogen, Carlsbad, CA, USA). Specific transcripts were amplified with gene-specific forward and reverse primers (see Table S2 in Additional file 4) in a Robocycler Gradient 96 thermocycler (Stratagene, Stratagene, La Jolla, CA, USA). The housekeeping gene β-actin was used as control to normalize the gene-specific expression. PCR products were separated by gel electrophoresis on 1.8% agarose gel in 1× Tris-acetate-ethylenediamine tetraacetate buffer and visualized by ethidium bromide staining, and images were captured using a Biorad Gel documentation system.

### Neuronal differentiation of human umbilical cord blood MSCs

We used two different protocols for neuronal differentiation of hUCB-MSCs. The first was a four-step induction protocol consisting of exposure to a series of growth factors that push the MSCs towards a neuronal lineage [24]. Briefly, the cells were exposed for 3 days to Step-1 medium consisting of IMDM supplemented with FGF-2 (5 ng/ml), retinoic acid (0.5 μM) and 1 mM 2-mercaptoethanol, followed by 3 days in Step-2 medium consisting of IMDM supplemented with 1 mM cAMP and 100 μM AsA. The cells were further exposed to Step-3 medium consisting of IMDM supplemented with 10 μM hydrocortisone and 1 mM cAMP, followed again by 3 days in Step-4 medium consisting of IMDM supplemented with 20 ng/ml αFGF, 10 ng/ml Shh, 10 ng/ml brain-derived neurotrophic factor, 10 ng/ml nerve growth factor, 25 ng/ml vitronectin, 100 μM AsA, 0.1 mM 3-isobutyl-1-methylxanthine, 10 μM forskolin and 20 nM phorbol myristate acetate. For the second protocol involving direct neuronal differentiation, we used normal neuronal differentiation medium containing neurobasal medium supplemented with FGF-2 (10 ng/ml) and 1% FBS for 5 days.

### Statistical analysis

Data from all the experiments are represented as the mean ± standard deviation of triplicates (*n *= 3) from three different experiments. Statistical significance between groups was calculated by independent Student *t *test assuming equal variance. *P <*0.05 was considered statistically significant.

## Results

Our initial interest was to isolate MSCs from UCB and check their potential to transdifferentiate into neurons, but surprisingly we found significant variations between the isolated MSCs from different batches - this led us to further characterize these hUCB-MSCs. All of the clinical parameters analyzed were in correlation with previously reported optimal condition for blood banking [27]. In total we collected 45 UCB samples, nine of which generated MSCs that were further expanded and characterized. The overall efficiency of MSC isolation from UCB therefore appears to be 20%. The majority of these 20% UCBs had high yield of MSCs and originated from babies with a higher mean body weight and a decrease in gestation period. There are previous reports where the increase in cell yield was reported to correlate with an increase in baby's body weight and a decrease in gestational duration [28,29].

Interestingly, the most viable and expanded hUCB-MSC cells (hUCB-MSC SCB4) obtained for our study also matched these criteria (3.87 kg body weight and 36 weeks of gestation), compared with the mean body weight of 2.81 ± 0.45 kg and a gestation duration of 37.93 ± 1.30 weeks. For most of our experiments we used these MSCs that showed variation in their neurogenic differentiation potential, even though all of them expressed MSC surface markers. We also used an immortalized hUCB-MSC cell line (RCB 2080 from RIKEN) as a positive control to characterize the proliferation and differentiation potential of the expanded hUCB-MSCs.

### Immunophenotyping of expanded human UCB-MSCs confirms their MSC phenotype

**Figure 1:**
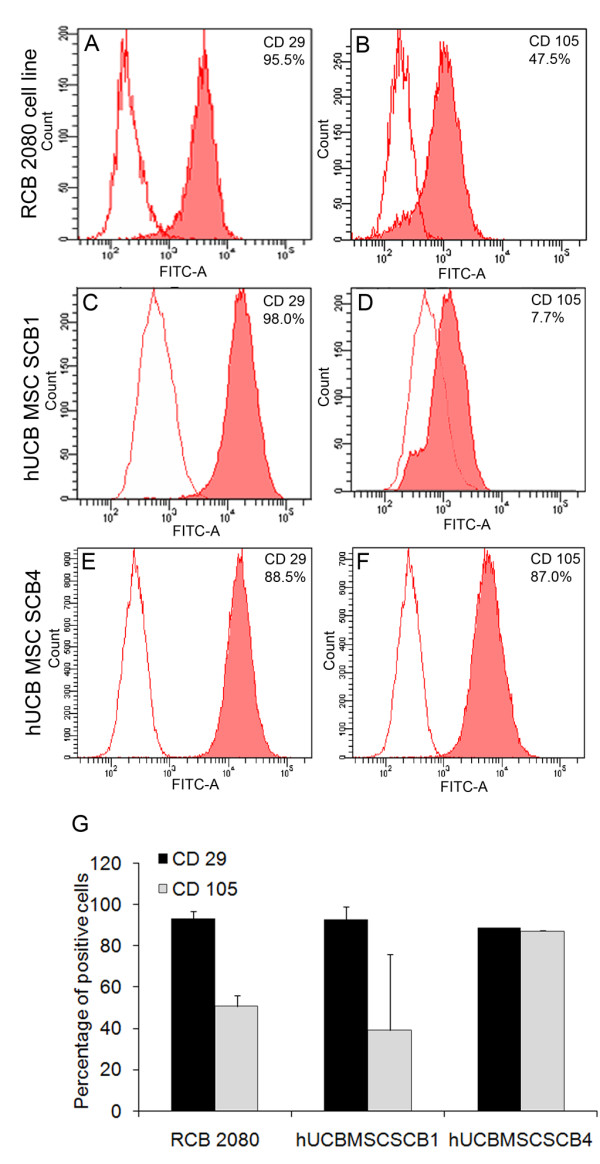
**Immunophenotyping of control RCB 2080 cell line and expanded human UCB-MSCs**. **(A) to (F) **Adherent human umbilical cord blood-derived mesenchymal stem cells (hUCB-MSCs) and the RCB 2080 cell line were trypsinized at passage number 3 and fluorescence-activated cell sorting (FACS) analysis was carried out with important MSC surface markers CD105 and CD29. All expanded hUCB-MSCs showed approximately 94% positivity for CD29 and 47% positivity for CD105. **(G) **Percentage of MSC-positive surface markers in the control RCB 2080 cell line and expanded hUCB-MSCs by FACS analysis. Blank histogram represents cells-only control. Data represented as mean ± standard deviation of triplicates (*n *= 3) from three different experiments.

To characterize the expanded hUCB-MSCs, we initially checked the expression profile of MSC-specific surface markers such as CD105, CD73, CD29 and CD44. Fluorescence-activated cell sorting analysis showed that all of the expanded hUCB-MSCs were positive for CD29 (88.5 ± 0.11%) and CD105 (87.2 ± 0.29%), although variation in percentage of CD105 expression was observed in few batches (Figure 1A to 1F). These results were compared with those of the immortalized hUCB-MSC cell line, which was also positive for CD29 (93.0 ± 3.56%) and CD105 (50.8 ± 5.21%) (Figure 1G). The variation in co-expression of CD29 and CD105 in different batches was the initial indication that the composition of all the expanded UCB-MSCs may not be similar even though all of them showed an adherent, fibroblastic morphology. Our results also indicated that all of the isolated hUCB-MSCs and the immortalized hUCB-MSC cell line were negative for hematopoietic surface markers CD34 and CD45 (data not shown).

**Figure 2:**
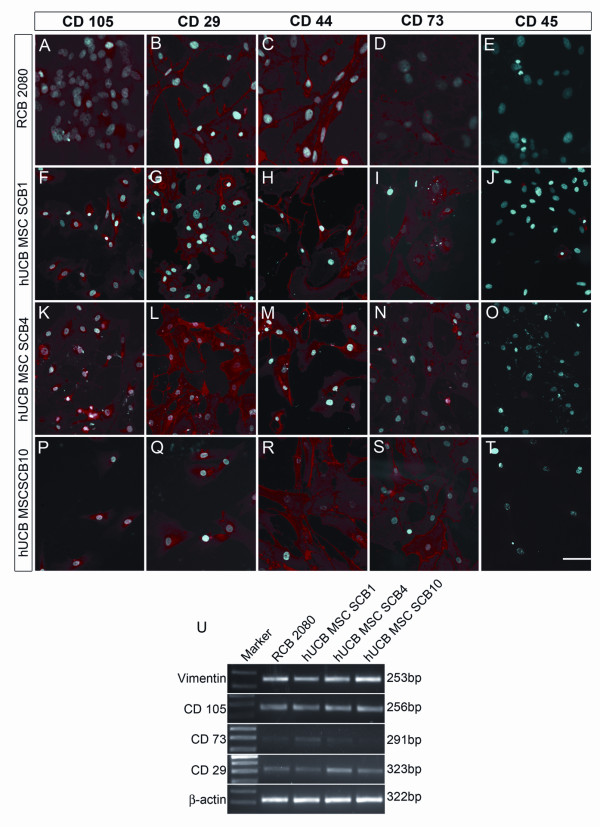
**Isolation of human UCB-MSCs and their characterization in comparison with the human UCB-MSC cell line**. **(A) to (T) **Immunofluorescence analysis of the control mesenchymal stem cells (MSC) cell line RCB 2080 and expanded human umbilical cord blood-derived (hUCB)-MSCs with MSC-positive surface markers CD105, CD29 and CD73 and MSC-negative surface marker CD45, clearly showing the RCB 2080 hUCB-MSC cell line as well as the expanded hUCB-MSCs staining positively for MSC-positive markers and negative for MSC-negative marker, confirming the MSC phenotype. **(U) **RT-PCR analysis of expanded proliferating hUCB-MSCs with MSC-positive surface markers such as CD29, CD73, CD105, and Vimentin showing good levels of expression at the mRNA level, also including the control hUCB-MSC cell line RCB 2080.

To further confirm the MSC phenotype of the isolated progenitors, we carried out immunocytochemical analysis with the abovementioned cell surface markers. Our results showed that majority of the expanded cells were positive for CD105, CD29, CD44 and CD73 and were negative for CD45 (Figure 2A to 2T). The MSC phenotype of these isolated progenitors was further corroborated with RT-PCR analysis with MSC-positive markers as mentioned above and also with Vimentin, another important MSC marker (Figure 2U).

### Unexpanded UCB-derived mononuclear cells express mesenchymal and pluripotency markers

We had previously observed a very high co-expression of CD29 (88.5 ± 0.11%) and CD105 (87.2 ± 0.29%) in the expanded hUCB-MSCs (hUCB-MSC SCB4; Figure 1E, F) compared with other batches and with the immortalized hUCB-MSC cell line (Figure 1A, B). We therefore decided to further characterize these progenitors with respect to expression of pluripotency markers.

**Figure 3:**
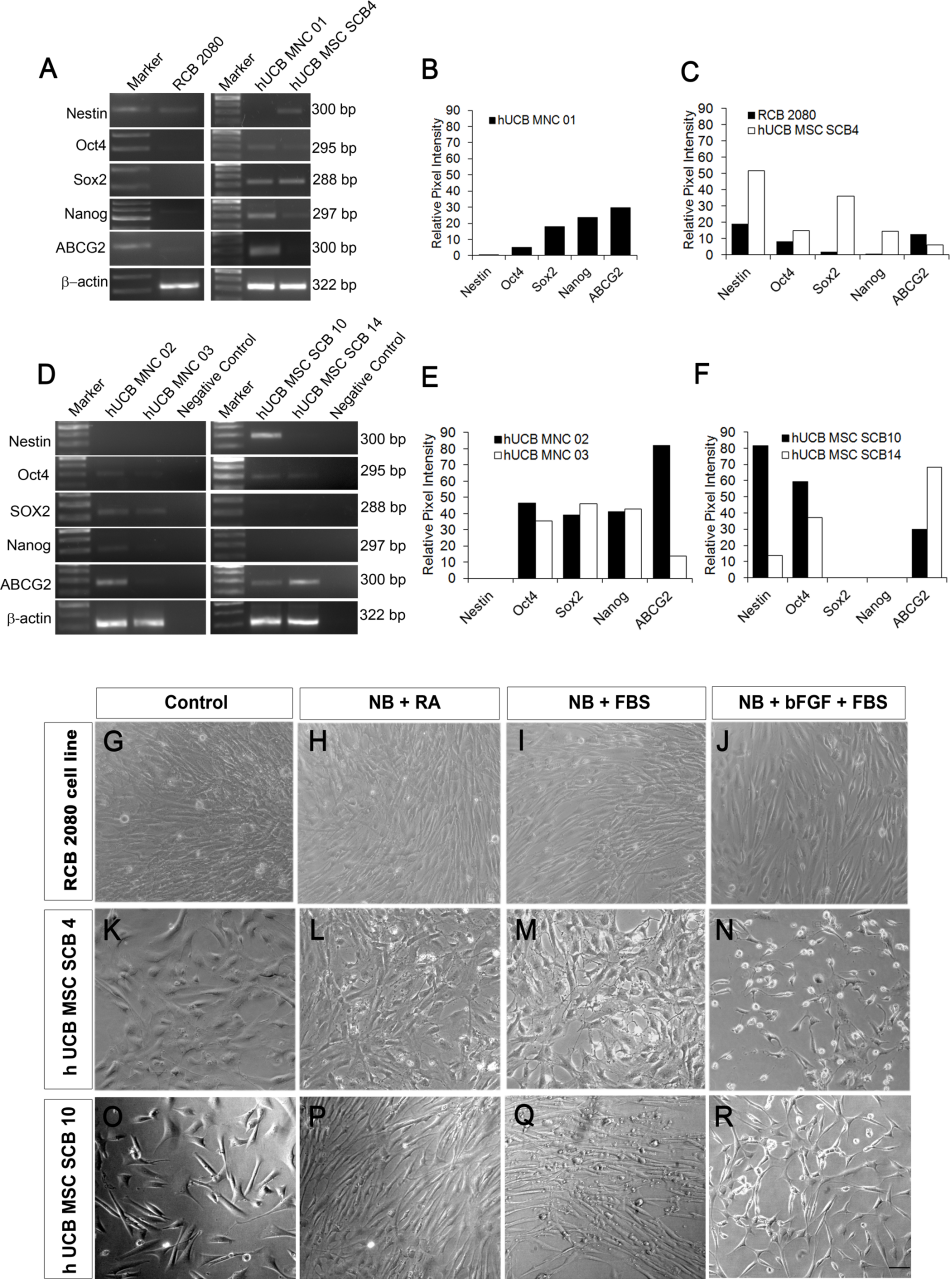
**Human UCB-MSCs show expression of pluripotent and neural progenitor markers**. **(A) **RT-PCR analysis shows the presence of transcripts corresponding to pluripotent as well as neural progenitor markers in freshly isolated human umbilical cord blood-derived (hUCB) mononuclear cell MNC01 and also in expanded mesenchymal stem cell (hUCB-MSC) SCB4 (lanes 4 and 5). hUCB MNC01 showed a significant level of expression of pluripotent markers but lacked nestin expression when compared with control RCB 2080 (lane 2), whereas hUCB-MSC SCB4 showed a higher level of nestin and reduced level of pluripotent marker expression. **(B) **Comparison of the relative pixel intensity of gene expression in hUCB MNC01. **(C) **Comparison of the relative pixel intensity of gene expression in RCB 2080 and hUCB-MSC SCB4. **(D) **Comparison of pluripotent marker and neural stem cell marker expression in two more sets of hUCB-MNCs (MNC02 and MNC03) and hUCB-MSCs (hUCB-MSC SCB10 and SCB14), which also shows a varying pattern of expression corroborating our previous observations that neural stem cell marker expression varies from sample to sample. **(E) **Relative pixel intensity of gene expression in hUCB MNC02 and MNC03. **(F) **Relative pixel intensity of gene expression in hUCB-MSC SCB10 and hUCB-MSC SCB14. **(G) to (R) **Treatment of control RCB 2080, hUCB-MSC SCB4 and hUCB-MSC SCB10 cells with neurobasal (NB) medium in combination with and without 0.5 μm retinoic acid (RA), 1% FBS and 10 ng basic fibroblast growth factor (bFGF). Scale bar = 100 μm.

RT-PCR analysis of hUCB-MSC SCB4 cells showed a very high expression level of nestin and Sox2 and moderate expression of Nanog and Oct4, and were negative for ABCG2, indicating a typical neural progenitor profile (Figure 3A, C). We also checked for expression of pluripotency markers in hUCB-MSC SCB10 and hUCB-MSC SCB14, and found that only hUCB-MSC SCB10 have high expression of nestin and Oct4, whereas hUCB-MSC SCB14 had a very low expression of nestin and Oct4 compared with hUCB-MSC SCB4 and hUCB-MSC SCB10 (Figure 3D, F). These results clearly show that the expression of nestin and other pluripotent markers vary in different batches of MSCs.

We next compared the expression profile of freshly isolated unexpanded hUCB-MNCs (hUCB MNC01, MNC02 and MNC03; Figure 3A, B, D, E) and found that MNC01 and MNC02 had high expression levels of pluripotency markers such as ABCG2, Sox2, Nanog and Oct4 but were negative for nestin. These results were very interesting since it appears that the MNCs isolated from UCB consists of progenitors expressing pluripotency markers, which later expand into phenotypically MSC-like cells expressing neural progenitor markers. Practically, we were unable to purify this population of progenitors expressing pluripotency markers from the freshly isolated MNCs since the percentage of these progenitors were very low. We also checked for a similar pluripotency/neural progenitor marker expression profile in the RCB 2080 cell line, and found that - except for the expression of nestin - all pluripotency/neural progenitor marker expression was low compared with the hUCB-MSC SCB4 and hUCB-MSC SCB10 progenitors (Figure 3A, C, D, F).

From our results it therefore appears that the hUCB-MSC SCB4 and hUCB-MSC SCB10 progenitors may have a very high neurogenic differentiation potential compared with other MSCs. To further confirm the neurogenic potential of hUCB-MSC SCB4 and hUCB-MSC SCB10 cells, we exposed them to the simple neuronal differentiation condition used for embryonic stem cell-derived neural progenitor differentiation and compared the expression with that of the RCB 2080 cell line [25]. To our surprise the hUCB-MSC SCB4 cells exposed to neurobasal medium and FBS (neurobasal + 1% FBS) and neurobasal + FGF-2 + 1% FBS readily differentiated into morphologically neuronal-like cells (Figure 3M, N), and hUCB-MSC SCB10 showed a moderate neuronal differentiation (Figure 3R) compared with RCB 2080 cells, which did not show any neuronal differentiation (Figure 3G to 3J). These results therefore confirmed that the hUCB-MSC SCB4 and hUCB-MSC SCB10 cells were different from the immortalized UCB-MSC cell line RCB 2080 with respect to its neurogenic potential. We further analyzed other batches of UCB-MSCs to check their potential to differentiate into neurons, but none of them showed neurogenic potential similar to hUCB-MSC SCB4 and hUCB-MSC SCB10.

### Percentage of proliferating BrdU^+ve^/nestin^+ve ^mesenchymal stem cells varies in different batches of umbilical cord blood

**Figure 4:**
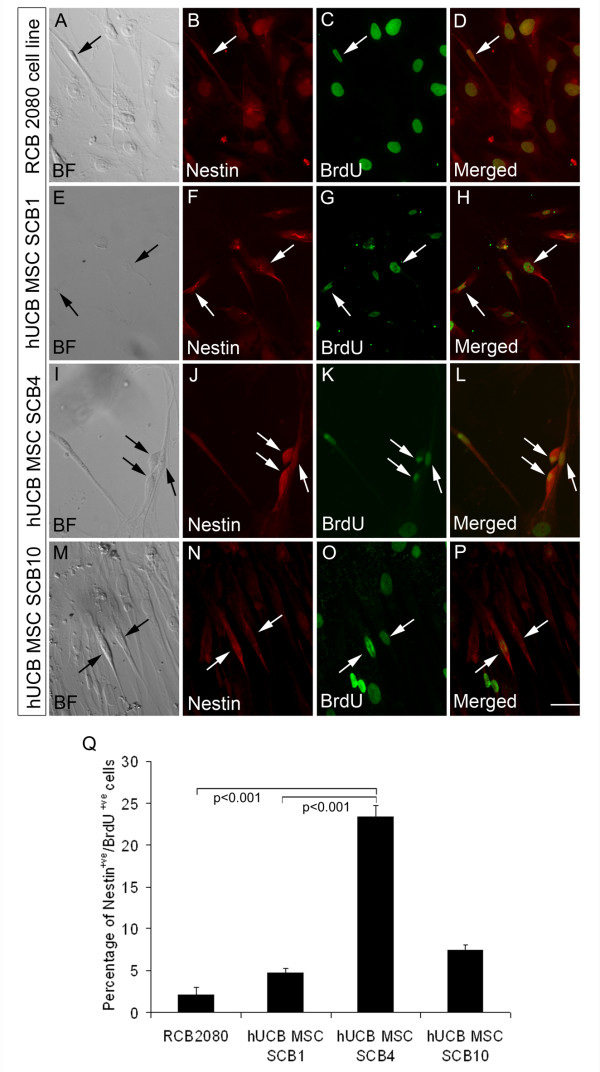
**Neural progenitor percentage present in isolated human UCB-MSCs varies in different human UCB samples**. **(A) to (P) **Immunofluorescence analysis with neural progenitor marker nestin and proliferation marker 5-bromo 2-deoxyuridine (BrdU) suggested the presence of proliferating neural progenitors in human umbilical cord blood-derived mesenchymal stem cells (hUCB-MSCs) and the number of nestin^+ve^/BrdU^+ve ^cells was different from batches to batches. **(Q) **Percentage of nestin^+ve^/BrdU^+ve ^cells in control hUCB-MSC cell line RCB 2080 and other expanded hUCB-MSCs SCB1, SCB4 and SCB10. The control RCB 2080 cell line showed only about 2% nestin^+ve^/BrdU^+ve ^cells, which was significantly low when compared with the expanded hUCB-MSCs. hUCB-MSC SCB4 showed a significantly high percentage of nestin^+ve^/BrdU^+ve ^cells (~23%), suggesting that these cells are more inclined to neuronal lineage. Another two batches, hUCB-MSC SCB1 and SCB10, also contained nestin^+ve^/BrdU^+ve ^cells but the percentage was less compared with the hUCB-MSC SCB4, and at the same time comparatively higher than the control hUCB-MSC cell line RCB 2080. Data represented as mean ± standard deviation of triplicates (*n *= 3) from three different experiments. Scale bar = 100 μm. BF, Bright Field.

Knowing that the hUCB-MSC SCB4 cells possess very high neurogenic potential, we next decided to compare the expression of neuroectodermal marker nestin in proliferating MSCs of other UCB batches and also with that of the immortalized RCB 2080 MSC cell line. For this comparison, different batches of cells were exposed to BrdU during expansion and the expression of neural progenitor marker nestin was analyzed by immunocytochemistry. Interestingly, the hUCB-MSC SCB4 cells had a very significantly high (23.33 ± 1.38, *P *< 0.001; Figure 4I to 4L, Q) percentage of cells having BrdU incorporation and co-expressing nestin compared with other batches, even though these cells showed co-expression of nestin and BrdU to a lesser extent (hUCB-MSC SCB1 and hUCB-MSC SCB10; Figure 4E to 4H, M to 4P, Q). The RCB 2080 cell line also showed a minimum number of BrdU^+ve ^and nestin^+ve ^MSCs (Figure 4A to 4D, Q).

**Figure 5:**
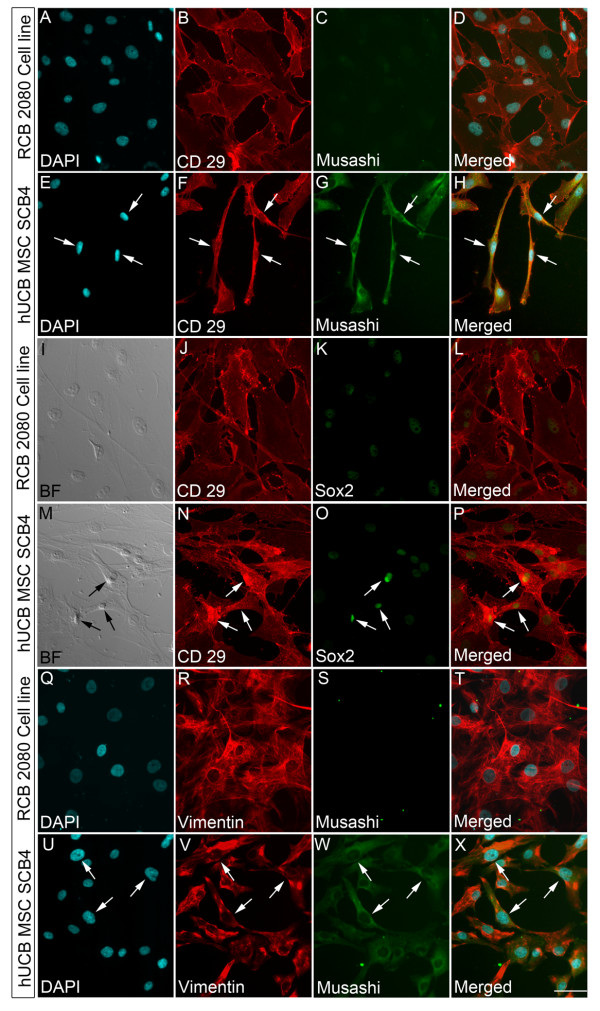
**Human UCB-MSC SCB4 express neural progenitor marker without losing their MSC identity**. **(A) to (H) **Expression of neural stem cell (NSC) marker Musashi was significantly high in human umbilical cord blood-derived mesenchymal stem cell (hUCB-MSC) SCB4 and co-expressed with CD29 when compared with RCB 2080 **(I) to (P)**. Although the RCB 2080 cell line showed the presence of the NSC marker Sox2, the expression was comparatively high in hUCB-MSC SCB4 and co-localized with CD44. **(Q) to (X) **Similar co-localization was also seen in hUCB-MSC SCB4 upon Vimentin-Musashi co-immunostaining, whereas co-localization was very low in control RCB 2080. Scale bar = 100 μm. DAPI, 4',6-diamidino-2-phenylindole.

To further confirm whether the progenitors expressing MSC markers phenotypically are those that also express neural progenitor markers, we tried to check for co-localization of both neural progenitor markers along with MSC surface markers. The comparison was made between the hUCB-MSC SCB4 and the RCB 2080 UCB-MSC cell line. Our results showed that majority of the CD29-expressing cells in hUCB-MSC SCB4 also co-expressed neural progenitor markers Musashi and Sox2 compared with the RCB 2080 cell line (Figure 5A to 5P). A similar result was also seen with MSCs co-expressing Vimentin and Musashi. Here, the percentage of Vimentin^+ve ^Musashi^+ve ^cells was again higher in hUCB-MSC SCB4 cells compared with the RCB 2080 cell line (Figure 5Q to 5X). These results further confirm that indeed there exist progenitors that phenotypically express MSC markers and also co-express neural progenitor markers in addition to progenitors positive for MSC markers alone.

A similar comparison was made with hUCB-MSC SCB10 cells where we could not find a significant co-localization of CD29 with Musashi, but were able to find cells co-localizing CD29 and Sox2 (see Figure S3 in Additional file 5). hUCB-MSC SCB4 and hUCB-MSC SCB10 therefore appear to have a higher neurogenic potential compared with hUCB-MSC SCB1, but with varying levels. These results confirmed that indeed there exists variation in the neurogenic potential among different batches of hUCB-MSCs.

### Human UCB-MSCs expressing neural progenitor markers instantaneously differentiate into neurons upon exposure to regular neuronal differentiation conditions

Since our previous results proved that the hUCB-MSC SCB4 cells have an inherent neurogenic potential, we next subjected them to a previously reported transdifferentiation condition [24]. We also included hUCB-MSC SCB10 cells since they have also shown higher expression of the neural progenitor marker nestin (Figure 3C, D).

**Figure 6:**
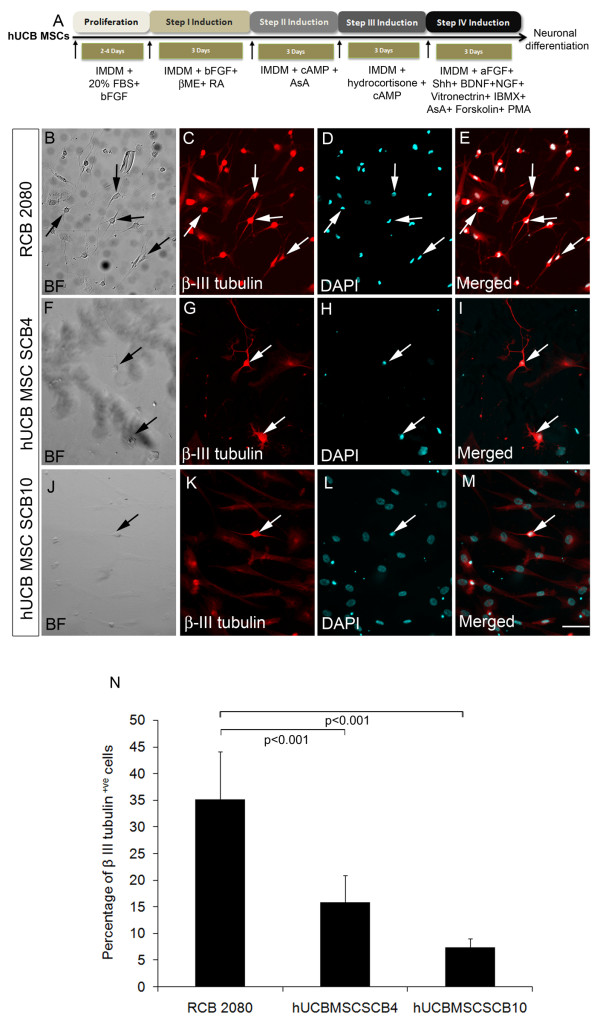
**Neuronal differentiation of human UCB-MSCs in four-step neuronal induction medium**. **(A) **Schematic of four-step neuronal induction condition for human umbilical cord blood-derived mesenchymal stem cell (hUCB-MSC) differentiation. **(B) to (E) **Differentiation of the control RCB 2080 hUCB-MSC cell line with the four-step induction protocol showed transdifferentiation of MSCs to neurons as ascertained by β-III tubulin immunostaining. **(F) to (I) **β-III tubulin immunostaining of neurons transdifferentiated from hUCB-MSC SCB4 cells with the four-step neuronal induction protocol. **(J) to (M) **Immunostaining of β-III tubulin, a neuronal marker on neurons from hUCB-MSC SCB10 upon four-step medium treatment. **(N) **Comparison of percentage of β-III tubulin-positive cells in control RCB 2080 with hUCB-MSC SCB4 and hUCB-MSC SCB10: ~35% β-III tubulin-positive cells were in RCB 2080, whereas ~15% cells in hUCB-MSC SCB4 and 7.4% cells in hUCB-MSC SCB10 were positive for β-III tubulin - showing a significant difference in pattern of differentiation between the hUCB-MSC cell line RCB 2080 and primary hUCB-MSC SCB4 and SCB10, indicating that neuronal differentiation potential of primary hUCB-MSCs vary from batch to batch. Data represented as mean ± standard deviation of triplicates (*n *= 3) from three different experiments. Scale bar = 100 μm. aFGF, acidic fibroblast growth factor; BF, Bright Feild; bFGF, basic fibroblast growth factor; βME, beta-mercaptoethanol; DAPI, 4',6-diamidino-2-phenylindole; IBMX, 3-isobutyl-1-methylxanthine; IMDM, Iscove's modified Dulbecco's medium; NGF, nerve growth factor; PMA, phorbol myristate acetate.

The MSCs were exposed to four different combinations of growth factors as described in Materials and methods (Figure 6A). The differentiation potential of hUCB-MSC SCB4 and hUCB-MSC SCB10 cells were compared with that of the RCB 2080 cell line. Our results indicate that the RCB 2080 cell line could be very effectively transdifferentiated into neurons and stained positive for β-III tubulin (35.16 ± 8.22; Figure 6B to 6E, N). The percentage of β-III tubulin-positive cells were also significantly high (*P *< 0.001) compared with hUCB-MSC SCB4 and hUCB-MSC SCB10 (15.90 ± 4.28 and 7.41 ± 1.59, respectively; Figure 6F to 6M, N). This was a very interesting observation since we expected hUCB-MSC SCB4 cells to generate more neurons compared with the RCB 2080 cell line. This result could be due to the fact that the RCB 2080 cell line expressing MSC markers alone was induced to transdifferentiate into neurons by the four-step induction condition, whereas hUCB-MSC SCB4 and hUCB-MSC SCB10 contained only a few cells that express MSC markers alone. Only these few cells could therefore have been transdifferentiated into neurons under these conditions. Ideally, however, the cells co-expressing MSC and neural progenitor markers could also have been differentiated into neurons with this condition, which is not the case here. Further experiments needs to be carried out to confirm this.

**Figure 7:**
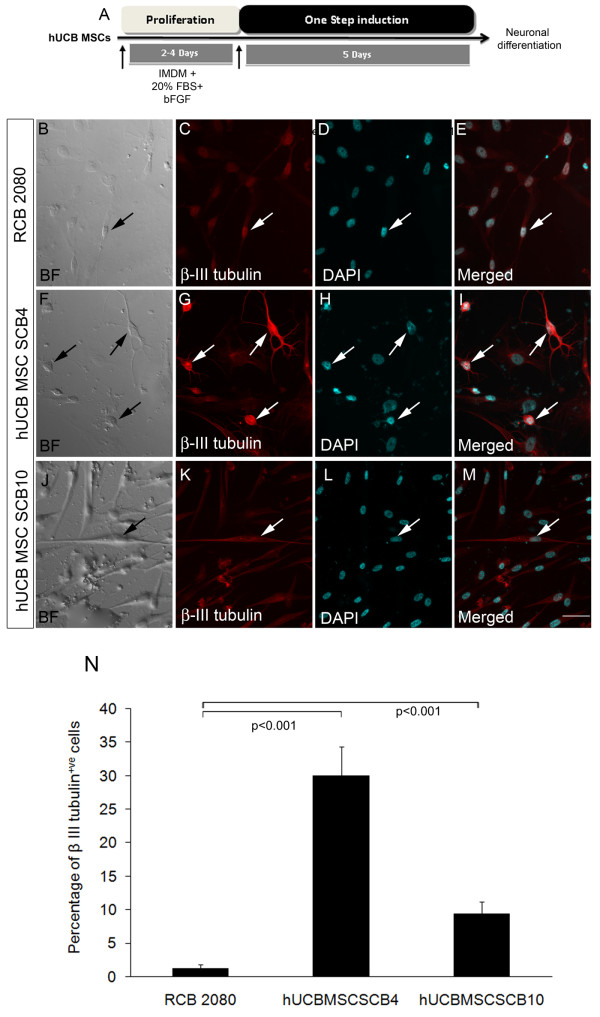
**Neuronal differentiation of human UCB-MSCs in one-step neuronal induction medium**. **(A) **Schematic of one-step neuronal induction condition for human umbilical cord blood-derived mesenchymal stem cell (hUCB-MSC) differentiation. After one-step neuronal induction, hUCB-MSC SCB4 generated more β-III tubulin^+ve ^neuronal-like cells **(F) to (I) **compared with the control MSC cell line RCB 2080 **(B) to (E)**. **(J) to (M) **β-III tubulin^+ve ^cells observed in the case of hUCB-MSC SCB10 but less compared with hUCB-MSC SCB4, indicating a variation in differentiation potential among hUCB-MSC samples. **(N) **Comparison of percentage of β-III tubulin-positive cells in control RCB 2080 with hUCB-MSC SCB4 and hUCB-MSC SCB10. Only ~4% β-III tubulin-positive cells were in RCB 2080, whereas ~30% cells in hUCB-MSC SCB4 and 9.37% cells in hUCB-MSC SCB10 were positive for β-III tubulin. Data are expressed as mean ± standard deviation of triplicates (*n *= 3) from three different experiments. Scale bar = 100 μm. BF, Bright Field; bFGF, basic fibroblast growth factor; DAPI, 4',6-diamidino-2-phenylindole; IMDM, Iscove's modified Dulbecco's medium.

We next exposed both the hUCB-MSC SCB4 and hUCB-MSC SCB10 and the RCB 2080 cell line to simple neuronal differentiation conditions for 5 days (Figure 7A). We observed a very significantly high percentage (30.08 ± 4.26, *P *< 0.001) of β-III tubulin-expressing cells in hUCB-MSC SCB4 and moderately high neuronal differentiation in hUCB-MSC SCB10 (9.37 ± 1.84), compared with the RCB 2080 cell line (1.22 ± 0.61; Figure 7B to 7M, N). These results further indicate that the RCB 2080 cell line, which has a typical MSC phenotype, may be transdifferentiating alone as a result of pressure from the combination of growth factors, whereas the hUCB-MSC SCB4 and hUCB-MSC SCB10 cells co-expressing MSC and neural progenitor markers, with inherent neurogenic potential, appear to easily differentiate into neurons without much pressure or induction with the combination of growth factors. Even though hUCB-MSC SCB10 has a higher neurogenic potential compared with the RCB 2080 cell line, the potential is still less compared with hUCB-MSC SCB4. This clearly shows that there is indeed variation in neurogenic potential between different batches of expanded MSCs.

## Discussion

MSCs isolated from UCB and bone marrow are shown to have an inherent potential to differentiate along the mesodermal lineage such as adipogenic, chondrogenic and osteogenic cells [30,31]. A large literature demonstrates the potential of hUCB-MSCs to transdifferentiate into neurons by inducing the cells to cross the germline barrier and generate an ectodermal specified lineage [24]. The UCB-MSC-derived neurons can be used for treating neurodegenerative disease and can be easily obtained from discarded cord blood. Recent reports have also shown, however, that the percentage of robust rapidly expanding MSCs obtained from UCB is very low [32,33]. Moreover, different approaches have been taken for generating neurons from UCB-MSCs. Jang and colleagues have demonstrated that treating MSCs with β-mercaptoethanol and retinoic acid resulted in a very drastic difference in cellular morphology of MSCs from a fibroblastic to a spindle-shaped elongated process resembling the neuronal phenotype [19]. Other studies have shown that the hUCB-MSCs need exposure to four-step treatment with a combination of growth factors for transdifferentiation into neurons [24]. These results together therefore indicate that there appears to be a large difference in the neurogenic potential of hUCB-MSCs which needs to be properly addressed. Our results have shown a lot of variation among the hUCB-MSCs obtained with respect to their neurogenic potential even though all of them express MSC surface markers.

The variation in neurogenic potential of the MSCs obtained seems to originate from the stage of UCB isolation; that is, the gestational period and body weight of the baby. Our preliminary observations have shown a direct correlation between the weight of the baby and the gestation period with respect to MSC yield (Table S1 in Additional file 1). The majority of UCB that gave a high yield of MSCs and had very high neuronal differentiation potential (hUCB-MSC SCB4) originated from babies with a higher mean body weight and a decreased gestation period. Previous reports have also shown a high yield of MSCs from babies with similar clinical parameters [28,29]. Although this observation is preliminary, it shows a positive correlation and needs to be investigated further.

**Figure 8:**
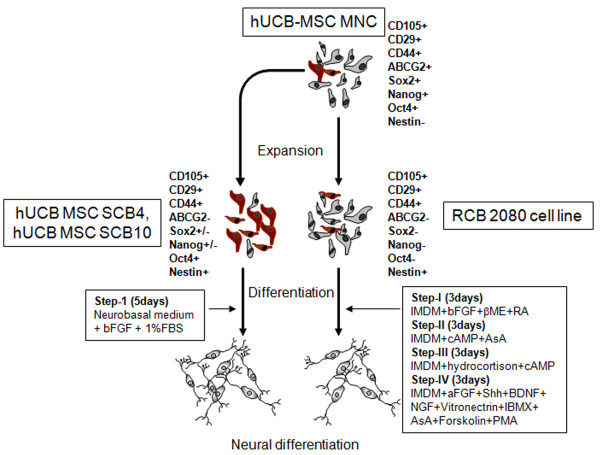
**Schematic showing the heterogeneity of mesenchymal population obtained from human umbilical cord blood**. Human umbilical cord blood-derived mononuclear cells (hUCB-MNCs) consist of a heterogeneous pluripotent population of cells with varying neurogenic potential. Upon expansion, the number of cells with inherent neurogenic potential may increase in some cases, which generates more neuronal differentiation instantaneously, while in other cases cells with less neurogenic potential are expanded, which requires extensive treatment with a combination of multiple growth factors to achieve neuronal differentiation. aFGF, acidic fibroblast growth factor; bFGF, basic fibroblast growth factor; βME, beta-mercaptoethanol; IBMX, 3-isobutyl-1-methylxanthine; IMDM, Iscove's modified Dulbecco's medium; MSC, mesenchymal stem cell; NGF, nerve growth factor; PMA, phorbol myristate acetate; RA, retinoic acid.

Another interesting observation was the expression of pluripotent stem cell markers in MNCs directly isolated from cord blood. These cells had a consistent expression of Oct4, a master regulator of pluripotency [34]. Oct4 has been previously reported to be involved in maintenance of stemness and multipotency of hUCB-MSCs [35] and also in induction of neurogenesis [36]. Our results showed a significant expression of ABCG2, Nanog and Sox2, confirming the pluripotent nature of hUCB-MNCs. Similar observation about pluripotent marker expression in UCB-MSCs had been reported previously by a few groups [21,37-39]. These results indeed suggest that there exists a small unique subpopulation of pluripotent progenitors in cord blood, which upon expansion generates MSCs with varied neuronal differentiation potential. Overall, our results have shown clearly that the unexpanded initially isolated MNCs have a subpopulation of MSC-like cells that are negative for nestin and positive for pluripotent stem cell markers Oct4, ABCG2, Nanog and Sox2 in addition to MSC markers CD105, CD29 and CD44 (Figure 8). These MNCs upon expansion generate MSCs that are positive for neuronal progenitor/pluripotency markers nestin and Oct4, but will be positive or negative for Sox2 and Nanog (high or moderate neurogenic potential; for example, hUCB-MSC SCB4 and hUCB-MSC SCB10, respectively; Figure 8) or negative for Oct4, Sox2 and Nanog (low neurogenic potential; for example, RCB2080 cell line). The progenitors expressing Oct4 may therefore be responsible for the high neurogenic potential.

Previous reports have also demonstrated the role of Oct4 in inducing neurogenesis [36,40]. If the number of the initial Oct4-expressing population is high then these cells may be preferentially expanded and will generate MSCs with high neurogenic potential, which can be very easily differentiated into neurons with minimal exposure to neurogenic factors (neurobasal medium with 1% FBS and FGF-2). On the contrary, if the initial Oct4-expressing population is low then the expanded MSCs will have very low number of cells with neurogenic potential. These MSCs would then require extensive exposure to a combination of growth factors to transdifferentiate the MSCs into neurons. This difference may be the reason why the RCB 2080 immortalized MSC line required extensive exposure to different combination of growth factors for neural differentiation, since this cell line was clonally generated from immortalized MSCs that lack the pluripotent population.

## Conclusion

Our results suggest that the expanded UCB-derived MSCs harbor a small unique population of cells that express pluripotent stem cell markers along with MSC markers and possess an inherent neurogenic potential. These latter pluripotent progenitors generate cells expressing neural progenitor markers and are responsible for the instantaneous neuronal differentiation upon exposure to regular neuronal differentiation conditions. We have also shown that the percentage of cells expressing neural progenitor markers may vary from batch to batch and the batches that have progenitors expressing MSC markers alone require extensive exposure to a combination of growth factors to transdifferentiate the MSCs into neurons. Our findings therefore form the basis for developing better neuronal differentiation protocols from MSCs, which can be used for therapeutic applications.

## Abbreviations

BrdU: 5-bromo 2-deoxyuridine; BSA: bovine serum albumin; FBS: fetal bovine serum; FGF: fibroblast growth factor; FITC: fluorescein isothiocyanate; hUCB: human umbilical cord blood; IMDM: Iscove's modified Dulbecco's medium; MNC: mononuclear cell; MSC: mesenchymal stem cell; PBS: phosphate-buffered saline; PCR: polymerase chain reaction; PE: phycoerythrin; RT: reverse transcriptase; Shh: Sonic Hedgehog; UCB: umbilical cord blood.

## Competing interests

The authors declare that they have no competing interests.

## Authors' contributions

MSD carried out the experimental design, and performed neuronal differentiation experiments, data analysis and manuscript preparation. GER was involved in sample collection, clinical parameter analysis and sample processing. TSD was involved in immunofluorescence analysis and quantification of Figure 4. TRS performed fluorescence-activated cell sorting analysis of Figure 1. VAR was involved in carrying out RT-PCR analysis of Figure 3. KEE was involved in interpretation of clinical parameters of the UCB samples collected. JJ was involved in experimental design, coordination of the study, interpretation of data and manuscript preparation. RMP was involved in coordination and interpretation of the data. All authors read and approved the manuscript for publication.

## Supplementary Material

Additional file 1 **Table S1 presenting characteristics of UCB samples that generated MSCs**. Click here for file

Addition file 2 **Figure S1 showing immunostaining of embryonic day 14 mouse cortical neuronal culture as a positive control for Nestin, Sox2 and Musashi antibodies, which confirms the specificity of antibodies used in our study**. Click here for file

Additional file 3 **Figure S2 showing immunostaining of hUCB-MSCs with secondary antibody alone, which shows that there is no background or nonspecific staining**. Click here for file

Additional file 4 **Table S2 presenting primer sequences and PCR conditions used for RT-PCR analysis**. Click here for file

Additional file 5 **Figure S3 showing co-localization of neural stem cell (NSC) and MSC markers in hUCB-MSC SCB10**. (A) to (F) CD29 and CD44 co-localization with NSC marker Musashi was comparably low in this batch of cells. (G) to (L) At the same time, hUCB-MSC SCB10 showed expression of Sox2 along with MSC markers CD29 and CD44, even though the number of Sox2-positive cells was low. Click here for file
