# Supplementary material for: Umbilical cord blood-derived mesenchymal stem cells consist of a unique population of progenitors co-expressing mesenchymal stem cell and neuronal markers capable of instantaneous neuronal differentiation
Source: Stem Cell Res Ther. 2012 Dec 19;3(6):57. doi: 10.1186/scrt148 (PMC3580487; doi:10.1186/scrt148)
Supplement: Additional file 1 — Table S1 presenting characteristics of UCB samples that generated MSCs. [file scrt148-S1.PDF]

Additional File-1

Supplementary Table-1:

Characteristics of UCB samples that generated MSCs

SI No.	Code No.	GESTL AGE(Wks)	PLACENTAL WT (g)	CORD LENGTH (cm)	SEX OF BABY	BABY WT (Kg)	BLOOD GROUP
1	hUCB MSC SCB1	38	534	57	F	3.32	A+
2	hUCB MSC SCB4	36	546	47	F	3.87	B+
3	hUCB MSC SCB 10	38	517	54	F	3.21	AB+
4	hUCB MSC SCB 12	37	497	57	F	2.98	A+
5	hUCB MSC SCB 13	38	498	53	F	2.98	B+
6	hUCB MSC SCB 14	37	528	55	M	3.65	A+
7	hUCB MSC SCB 15	38	543	52	F	3.67	AB+
8	hUCB MSC SCB 16	37	528	52	M	3.24	O+
9	hUCB MSC SCB 17	36	485	54	M	2.48	B+
