# Supplementary material for: Umbilical cord blood-derived mesenchymal stem cells consist of a unique population of progenitors co-expressing mesenchymal stem cell and neuronal markers capable of instantaneous neuronal differentiation
Source: Stem Cell Res Ther. 2012 Dec 19;3(6):57. doi: 10.1186/scrt148 (PMC3580487; doi:10.1186/scrt148)
Supplement: Addition file 2 — Figure S1 showing immunostaining of embryonic day 14 mouse cortical neuronal culture as a positive control for Nestin, Sox2 and Musashi antibodies, which confirms the specificity of antibodies used in our study. [file scrt148-S2.PDF]

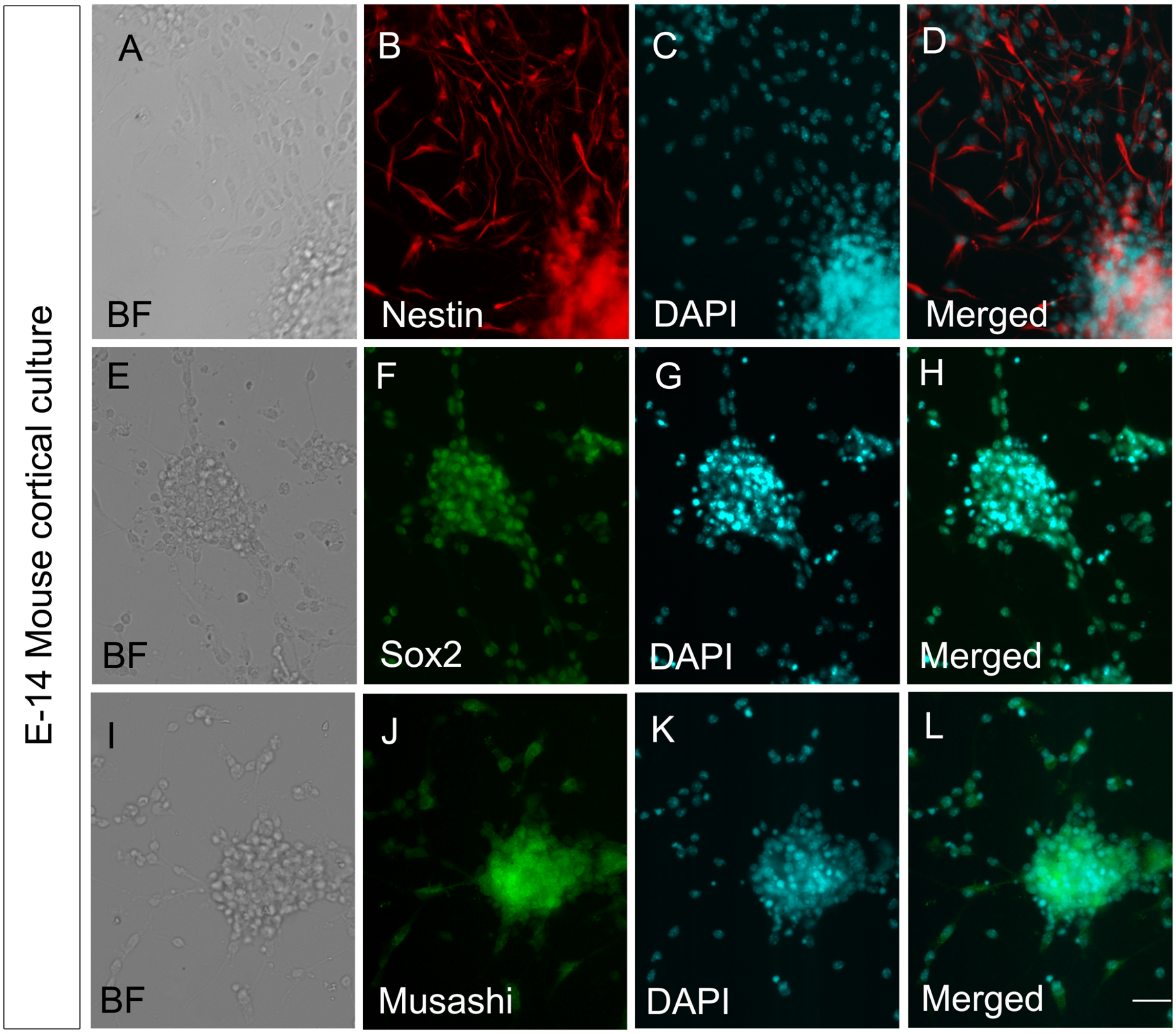

Addition File-2, Figure S1. Immunostaining of Embryonic day 14 mouse cortical neuronal culture as a positive control for Nestin, Sox2 and Musashi antibodies which confirms the specificity of antibodies used in our study.
