# Supplementary material for: Umbilical cord blood-derived mesenchymal stem cells consist of a unique population of progenitors co-expressing mesenchymal stem cell and neuronal markers capable of instantaneous neuronal differentiation
Source: Stem Cell Res Ther. 2012 Dec 19;3(6):57. doi: 10.1186/scrt148 (PMC3580487; doi:10.1186/scrt148)
Supplement: Additional file 3 — Figure S2 showing immunostaining of hUCB-MSCs with secondary antibody alone, which shows that there is no background or nonspecific staining. [file scrt148-S3.PDF]

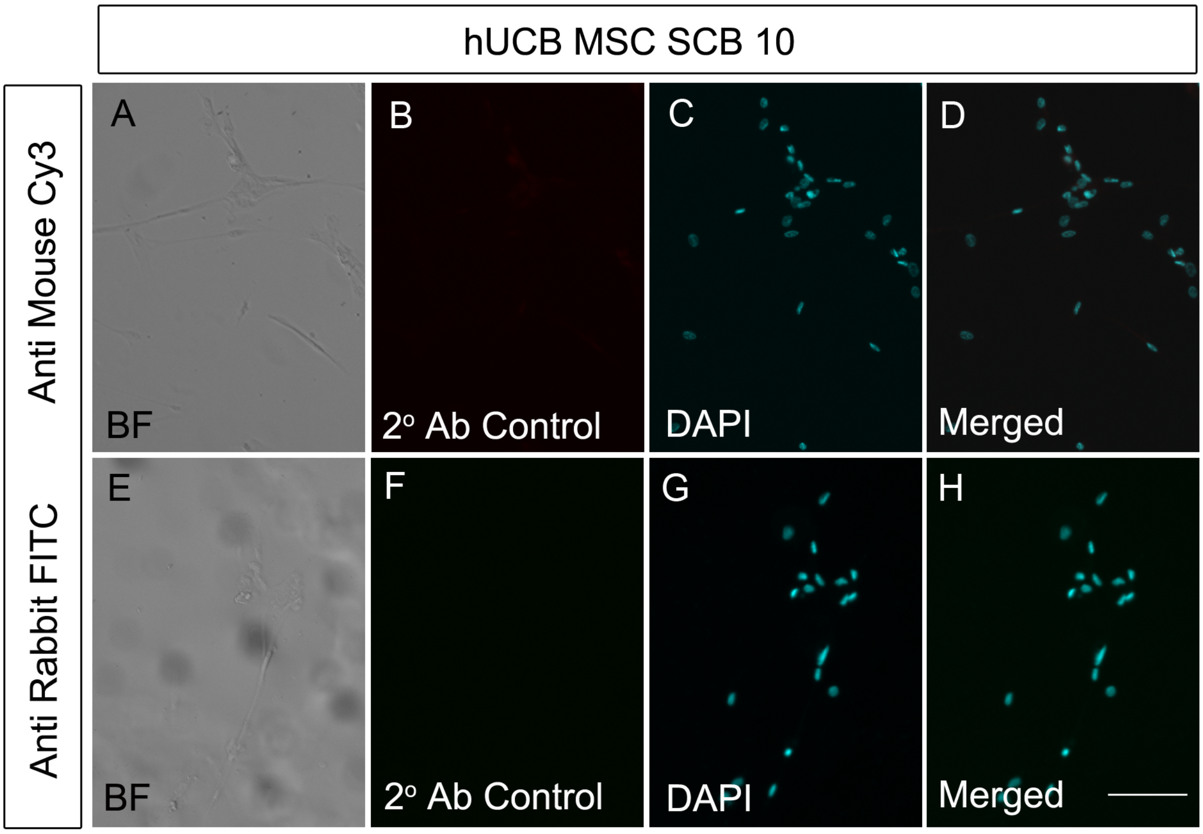

Additional File-3, Figure S2. Immunostaining of hUCB MSCs with secondary antibody alone which shows that there is no background or non-specific staining.
