# Supplementary material for: Umbilical cord blood-derived mesenchymal stem cells consist of a unique population of progenitors co-expressing mesenchymal stem cell and neuronal markers capable of instantaneous neuronal differentiation
Source: Stem Cell Res Ther. 2012 Dec 19;3(6):57. doi: 10.1186/scrt148 (PMC3580487; doi:10.1186/scrt148)
Supplement: Additional file 4 — Table S2 presenting primer sequences and PCR conditions used for RT-PCR analysis. [file scrt148-S4.PDF]

Additional File-4

Supplementary Table-2: Primer sequences and PCR conditions used for RT-PCR analysis

Gene name	Primer sequence	Ann temp. (^{o}C)	Product size (bp)	Accession no.
**β-actin**	F 5' AGACTTCGAGCAGGAGATG 3'<br>R 5' CTTGATCTTCATGGTGCTAGG 3'	56	322	**NM_007393**
**CD 105**	F 5' CCAGCATTGTCTCACTTCAT 3'<br>R 5 ' AAGACAAACTTGTCACCCCT 3'	56	256	**NM_000118.2**
**CD 73**	F 5' CACTATCTGGTTCACCGTGT 3'<br>R 5' TTTGAGAGAAAAGGGGTTTC 3'	56	291	**NM_002526.3**
**CD 29**	F 5' CGCGGAAAAGATGAATTT 3'<br>R 5' TCTCTGCTGTTCCTTTGCTA 3'	56	323	**NM_002211.3**
**Vimentin**	F 5' GAGAGAGGAAGCCGAAAAC 3'<br>R 5' CACACTTTCATATTGCTGACG 3'	56	253	**NM_003380.3**
**Oct4**	F 5' GGAGCTAGAACAGTTTGCC 3'<br>R 5' CCTCACACGGTTCTCAATG 3'	56	295	**NM_001173531.1**
**Nanog**	F 5' AATGAAATCTAAGAGGTGGCAG 3'<br>R 5' TCCTCTCCACAGTTATAGAAGG 3'	56	297	**NM_024865.2**
**Sox2**	F 5' CCAAGACGCTCATGAAGAAG 3'<br>R 5' TGGTCATGGAGTTGTACTGC 3'	60	288	**NM_003106.3**
**ABCG2**	F 5' AGCAGGGACGAACAATCATC 3'<br>R 5' CCTGCTTGGAAGGCTCTATG 3'	56	300	**NM_004827.2**
**Nestin**	F 5' GGATCAGATGACATTAAGACCC 3'<br>R 5' TCCAGTGGTTCTTGAATTTCC 3'	56	300	**NM_006617.1**
