# Supplementary material for: Umbilical cord blood-derived mesenchymal stem cells consist of a unique population of progenitors co-expressing mesenchymal stem cell and neuronal markers capable of instantaneous neuronal differentiation
Source: Stem Cell Res Ther. 2012 Dec 19;3(6):57. doi: 10.1186/scrt148 (PMC3580487; doi:10.1186/scrt148)
Supplement: Additional file 5 — Figure S3 showing co-localization of neural stem cell (NSC) and MSC markers in hUCB-MSC SCB10. (A) to (F) CD29 and CD44 co-localization with NSC marker Musashi was comparably low in this batch of cells. (G) to (L) At the same time, hUCB-MSC SCB10 showed expression of Sox2 along with MSC markers CD29 and CD44, even though the number of Sox2-positive cells was low. [file scrt148-S5.PDF]

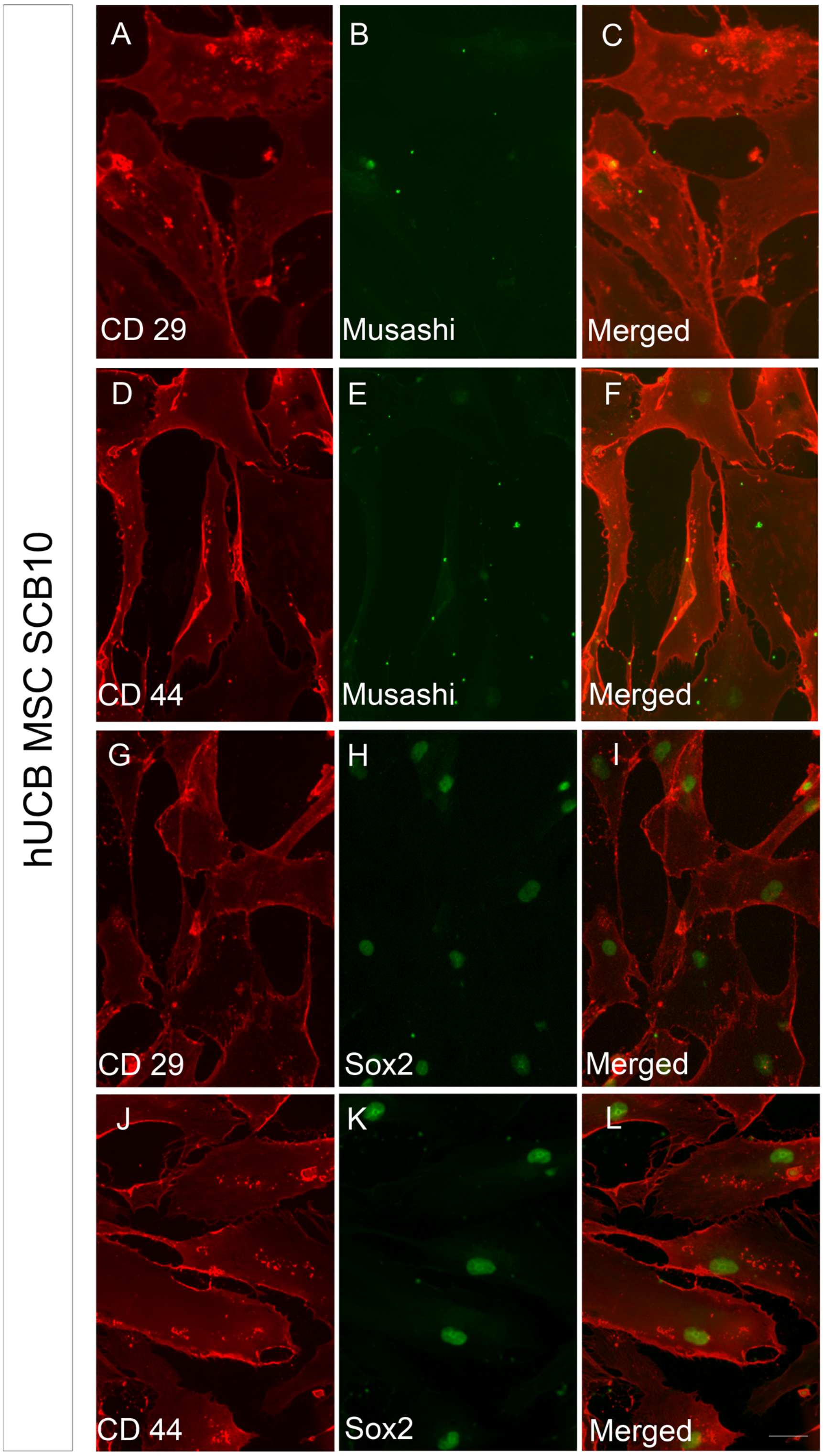

Additional File-5, Figure S3. Co-localization of NSC and MSC markers in hUCB MSC SCB10. (A-F) CD29 and CD44 co-localization with NSC marker Musashi was comparably low in this batch of cells. (G-L) At the same time, hUCB MSC SCB10 showed expression of Sox2 along with MSC markers CD29 and CD44, even though the number of Sox2 positive cells was low.
